# Frozen Elephant Trunk Procedure and Risk for Distal Stent-Graft-Induced New Entries

**DOI:** 10.1055/s-0042-1756666

**Published:** 2022-12-15

**Authors:** Maximilian Kreibich, Tim Berger, Bartosz Rylski, Matthias Siepe, Martin Czerny

**Affiliations:** 1 Department of Cardiovascular Surgery, University Heart Center Freiburg, University Hospital Freiburg, Freiburg, Germany; 2 Faculty of Medicine, University of Freiburg, Freiburg, Germany

**Keywords:** frozen elephant trunk, dSINEs, aortic dissection, stent-graft, aortic clinic

## Abstract

The frozen elephant trunk (FET) procedure is known as an effective treatment option for patients with any aortic pathology involving the aortic arch. However, there is growing evidence that many patients often require secondary intended, expected, or unexpected aortic reinterventions during follow-up. In those with underlying aortic dissection pathology, a substantial risk for developing distal stent-graft-induced new entries (dSINEs) has been identified as one cause for secondary aortic reinterventions. dSINE can develop at any time after the FET procedure. Endovascular treatment is generally feasible and safe to close the newly formed entry with low procedural risk. Nevertheless, all patients need continuous follow-up after FET treatment, ideally in a specialized aortic outpatient clinic.

## Introduction

Total arch replacement via the frozen elephant trunk (FET) technique has become a well-established treatment option for patients with acute or chronic aortic dissections (Type A, Type non-A and non-B, or Type B), those with aortic arch aneurysms, and even for patients with penetrating aortic ulcers in whom thoracic endovascular repair is unfeasible or unsafe. 1 2 3 4 5 6 There has also been a trend favoring the FET over isolated total arch replacement or conventional elephant trunk in recent years. Factors include the excellent proximal landing zone provided by the FET's stent-graft component, an anticipatory strategy regarding any progression of the underlying aortic pathology, FET's potential to reexpand the true lumen, and the ability to close proximal entries within the descending aorta. Hence, there is now solid European consensus, and many aortic centers now favor the FET for total arch replacement (“The elephant trunk is freezing”). 5 7 8

## Aortic Remodeling and Reinterventions

In patients with acute or chronic aortic dissection who underwent FET implantation, several groups demonstrated positive aortic remodeling of the descending and abdominal aorta. Such studies were recently published by Berger et al, Shrestha et al, and Dohle et al. 9 10 11

Despite their promising results, there is evidence of a significant risk of aortic reinterventions after FET implantation. 12 In competing risk regression analyses (competing risk: death), this risk rose to as high as 64% after 3 years. Note that one needs to differentiate between intended or expected secondary aortic reinterventions and those that are unexpected or unintended. 12

Young patients, especially, suffering from any type of chronic or chronic residual aortic dissection carry an inherent risk for aortic (re)interventions because of chronic diameter progression. 13 14 This risk still remains after the FET procedure. Nevertheless, the FET procedure is able to treat in one step the complete proximal aorta including the ascending aorta, aortic arch, and proximal descending aorta. It also provides an excellent platform for the dichotomous treatment of downstream aortic segments with comparably low risk. 6 15 16

Hence, in a relatively simple step, distal stent-graft elongation is possible up to the level of the celiac trunk following FET implantation. 16 In case this two-staged-approach fails to stabilize the remaining aorta, open thoracoabdominal aortic replacement is simplified because the anastomosis site has been moved distally. This procedure becomes comparable to a Crawford's type-4 scenario and is referred to as the three-step approach. 15 Without distal stent-graft extension as the second step, surgical treatment would amount to a more invasive Crawford's type-2 scenario. Lung compression can be significantly minimized in this third step when the anastomosis site is moved distally through an endovascular second step. 15

**Fig. 1:**
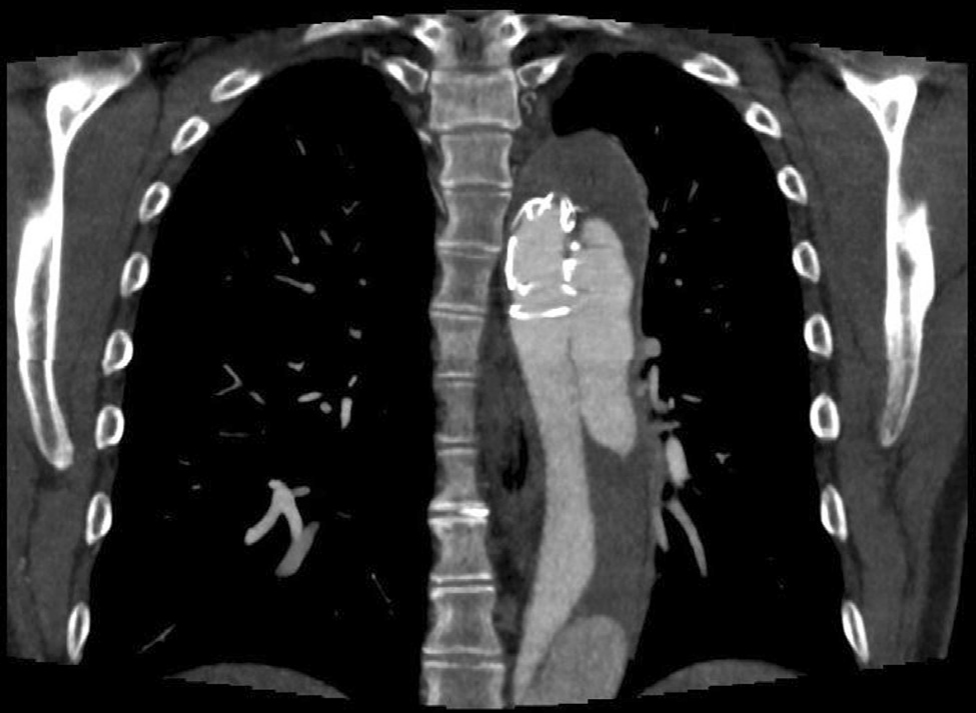
Representative computed tomographic scan of a distal stent-graft induced new entry (dSINE) that developed 1 year after frozen elephant trunk implantation.

For such conceptual, planned, or expected secondary aortic reinterventions following the FET, the “risk” designation seems inappropriate. Yet, there is also a substantial risk associated with unintended and unexpected aortic reinterventions following FET treatment. 12 Distal stent-graft-induced new entries (dSINEs) have been identified as one significant reason for such unintended secondary aortic reinterventions ( Fig. 1 ). 17

## Distal Stent-Graft-Induced New Entries following the Frozen Elephant Trunk Procedure

Dong et al defined a stent-graft-induced new entry as “a new tear” caused by a stent-graft regardless of natural disease progression or iatrogenic injury. 18 Because dSINE leads to false lumen perfusion, there is a significant risk for negative aortic remodeling being exacerbated to trigger acute aortic rupture. Hence, a dSINE occurrence should be considered as treatment failure. Because mortality rates for untreated dSINE are known to be as high as 25%, diagnosis of a dSINE should also represent a treatment indication. 19 Fortunately, dSINE therapy is quite simple via endovascular techniques. 16 17 19

The risk for developing a dSINE after the FET procedure was recently reported by our group to be up to 25% after 3 years. 17 The incidence of dSINE developing after the FET procedure is, thus, higher than the 6% incidence of dSINE development following conventional thoracic endovascular aortic repair. 20 Of note, dSINE may develop at any time following the FET procedure—even years after hybrid-graft implantation. 16 17

The interaction between a comparably stiff stent-graft and a flexible, thin dissection membrane seems to be responsible for dSINE development. 17 21 In chronic dissections, the dissection membrane loses elasticity and may become more likely to develop a dSINE. 21 22 23 Yet, the acuity of the dissection was not identified as a risk factor for dSINE formation in our recent investigation. 17 To prevent dSINE, the stent-graft component should not be oversized. While oversizing is necessary in conventional endovascular stent-graft treatment to anchor the grafts, it is unnecessary in case of a FET implantation because the device itself functions as an immobile stent-graft anchor. Hence, the stent-graft simply needs to re-expand the true lumen via its radial force and sufficiently seal off the true lumen to prevent a type IB endoleak.

To select the correct stent-graft size in acute scenarios, we apply a method first described by Rylski et al 24 : we subtract 3 mm from the aortic diameter measured between the left carotid and left subclavian artery, we add 8 mm to the true lumen diameter in the dissected aorta's first quartile or we measure the maximum diameter of the dissected true lumen diameter at the level of the anticipated FET stent-graft landing. In chronic scenarios, we recommend measuring the true lumen's circumference at the level of the anticipated FET stent-graft landing, always employing the smallest stent-graft sizes available. 12 In any case, one can always measure the true lumen's diameter by carefully inserting intraoperatively a dilator into the true lumen.

Potential risk factors for developing a dSINE following stent-graft placement are summarized in Table 1 . Note that no clinical risk factors have yet been identified for dSINE development in patients receiving a FET prosthesis. 17 Theoretically, a sharp angle between the stent-graft and native true lumen, particularly after implanting the FET device in zone 2 and/or using shorter stent-grafts, may increase the risk for dSINE formation (even though single-center studies have not proven this to be a risk factor 17 22 23 25 ). It seems plausible that placing the stent-graft within the native aortic arch or in the very proximal descending aorta causes a sharp angle between the stent-graft and the downstream descending aorta. In addition, when forcing a short stent-graft downward into the straight descending aorta, the stent-graft may potentially return to a straight alignment with the conventional arch component of the FET device causing large dSINE as recently demonstrated by our group. 26 The same mechanisms may potentially be responsible for the higher dSINE incidence following the FET procedure in comparison to conventional Thoracic Endovascular Aortic Repair (TEVAR). Nevertheless, in our opinion, the advantages of a zone 2 implantation—namely easier and faster surgical implantation and a lower risk of spinal cord ischemia—still outweigh the theoretical disadvantage of dSINE formation. 17 27 28 After all, as long as patients are being routinely and closely followed, dSINE formation is an easy-to-detect complication. Hence, in our center, patients are routinely followed-up after 6 months, 12 months, and yearly thereafter. We perform computed tomography angiography scans before FET implantation, before discharge, during every follow-up visit, and whenever clinically warranted.

**Table 1:** Summary of potential risk factors for developing distal stent-graft induced new entries

*Shown in meta-analysis* :
Chronic aortic dissection
Oversizing/taper ratio
*Single center studies/own clinical experience* :
Short stent grafts
Sharp angle between the stent-graft and true lumen
Connective tissue disease

## Radial Force of the Frozen Elephant Trunk Stent-Grafts

Since no clinical risk factors for dSINE development following the FET procedure have been identified, and despite the fact that they occur in the absence of oversizing, the radial force of the FET stent-graft's distal end should be as low as possible to prevent injuries to the flexible dissection membrane. 17 Two FET prostheses are now on the European market: the Thoraflex (Ltd., Inchinnan, United Kingdom) and the E-Vita Open (Cryolife Jotec Inc., Hechingen, Germany). We demonstrated that the Thoraflex graft's distal stent-graft end is stiffer than the E-Vita Open graft's in ex vivo mechanical tests. 17 Moreover, when the grafts were confined, the Thoraflex graft became even stiffer. The relatively stiff, closed distal ring at the Thoraflex prosthesis stent-graft's distal end is probably responsible for this increased stiffness compared with the E-Vita graft's more flexible z-design. 17 However, we wish to emphasize that a large multicentric study relying on data from three large European aortic centers failed to reveal any statistically significant difference in the dSINE occurrence when comparing these two grafts. In fact, the numerical incidence was actually lower in patients treated with the Thoraflex graft (Thoraflex graft: 15% vs. E-Vita graft: 18%, *p* = 0.19). 29

## Distal Stent-Graft-Induced New Entries Treatment

Endovascular stent-graft extension to cover the newly formed entry is usually possible, and postoperative complications are rare. 16 17 In our center, we normally access the femoral arteries percutaneously using preclosure techniques, and we usually extend TEVAR down to the level of the thoracoabdominal transition in close proximity to the celiac trunk offspring. The FET stent-graft is our proximal landing zone, and we normally oversize proximally by 2 mm (most proximal stent-graft diameter to the stent-graft diameter of the FET). Distally, we avoid any oversizing and use the above-mentioned methods to calculate the diameter of the true lumen. 16 24 Because of the ideal artificial proximal landing zone of the FET graft, we follow this standardized management also in patients with connective tissue disease when the diameter threshold for the treatment of the descending aorta is met. Because these patients have an inherent high risk for negative distal aortic remodeling, they may potentially benefit most from the three-step-approach. 15

Note that recurring dSINE may form after distal stent-graft extension at the distal end of the newly implanted stent-graft. 20 Furthermore, to maximize spinal cord protection, we only carry out distal stent-graft extension after implanting a cerebrospinal fluid drainage the day before surgery. 5 8 For the same reason, we generally avoid concomitant distal stent-graft extension during the FET implantation. In case of planned intervention, we usually perform downstream TEVAR 6 months following FET implantation. When clinically necessary (e.g., in case of dSINE formation), we perform TEVAR as soon as possible.

## Conclusion

Although the FET procedure is an excellent treatment option for patients with aortic pathologies involving the aortic arch, surgeons need to be aware of the substantial rate of planned, anticipated, but also unplanned or unexpected aortic reinterventions. dSINEs are known to be one potential cause for aortic reinterventions, and they can develop at any time during follow-up after FET implantation. Thus, it is mandatory that all patients who have undergone the FET procedure be routinely followed-up, ideally in a highly specialized aortic clinic.
